# Supplementary material for: Hot spot prediction in protein-protein interactions by an ensemble system
Source: BMC Syst Biol. 2018 Dec 31;12(Suppl 9):132. doi: 10.1186/s12918-018-0665-8 (PMC6311905; doi:10.1186/s12918-018-0665-8)
Supplement: Supplementary file 2 — All relevant MATLAB code. A simple Matlab implement of our predictor. (ZIP 19,742 kb) [file 12918_2018_665_MOESM2_ESM.zip › Additional file 2/Read ASA/ASA website/BID/1DVAX.htm]

 NetSurfP - Protein Surface Accessibility and Secondary Structure Predictions

  

|  |  |  |
| --- | --- | --- |
|  |  | NetSurfP - Protein Surface Accessibility and Secondary Structure Predictions  Technical University of Denmark |

  


---

```
# For publication of results, please cite:
# A generic method for assignment of reliability scores applied to solvent accessibility predictions.
# Bent Petersen, Thomas Nordahl Petersen, Pernille Andersen, Morten Nielsen and Claus Lundegaard
# BMC Structural Biology 2009, 9:51 doi:10.1186/1472-6807-9-51
#
# Column 1: Class assignment - B for buried or E for Exposed - Threshold: 25% exposure, but not based on RSA
# Column 2: Amino acid
# Column 3: Sequence name
# Column 4: Amino acid number
# Column 5: Relative Surface Accessibility - RSA
# Column 6: Absolute Surface Accessibility
# Column 7: Z-fit score for RSA prediction
# Column 8: Probability for Alpha-Helix
# Column 9: Probability for Beta-strand
# Column 10: Probability for Coil
E A  1DVAX                  1    0.790  87.091  -0.560   0.003   0.003   0.994
E L  1DVAX                  2    0.394  72.196  -0.834   0.018   0.047   0.935
B C  1DVAX                  3    0.235  32.966  -1.620   0.018   0.088   0.893
E D  1DVAX                  4    0.681  98.161  -1.032   0.053   0.043   0.903
E D  1DVAX                  5    0.519  74.846  -0.669   0.058   0.017   0.925
E P  1DVAX                  6    0.494  70.127  -0.693   0.115   0.016   0.868
E R  1DVAX                  7    0.446 102.065   0.130   0.184   0.043   0.773
B V  1DVAX                  8    0.114  17.553  -0.162   0.191   0.086   0.723
E D  1DVAX                  9    0.370  53.389  -0.548   0.307   0.165   0.527
E R  1DVAX                 10    0.387  88.646   0.324   0.321   0.252   0.427
B W  1DVAX                 11    0.140  33.766   0.320   0.373   0.401   0.226
B Y  1DVAX                 12    0.201  42.996   0.765   0.373   0.401   0.226
B C  1DVAX                 13    0.080  11.204  -1.224   0.373   0.401   0.226
E Q  1DVAX                 14    0.471  84.103   0.776   0.268   0.505   0.227
B F  1DVAX                 15    0.251  50.416  -0.887   0.252   0.423   0.325
E V  1DVAX                 16    0.371  57.007   0.062   0.199   0.152   0.649
E E  1DVAX                 17    0.907 158.400   0.622   0.003   0.003   0.994

Explain the output.  Go back.
```

---
